# The *Arachno*‐Zintl Ion (Sn_5_Sb_3_)^3−^ and the Effects of Element Composition on the Structures of Isoelectronic Clusters: Another Facet of the Pseudo‐Element Concept

**DOI:** 10.1002/anie.202002863

**Published:** 2020-07-08

**Authors:** Robert J. Wilson, Florian Weigend, Stefanie Dehnen

**Affiliations:** ^1^ Fachbereich Chemie and Wissenschaftliches Zentrum für, Materialwissenschaften Philipps-Universität Marburg Hans-Meerwein Straße 4 35043 Marburg Germany; ^2^ Institute of Nanotechnology Karlsruhe Institute of Technology Hermann-von-Helmholtz-Platz 1 76344 Eggenstein-Leopoldshafen Germany

**Keywords:** binary Zintl clusters, DFT calculations, mass spectrometry, pseudo-element concept, X-ray diffraction

## Abstract

The pseudo‐element concept, in its most general formulation, states that isoelectronic atoms form equal numbers of bonds. Hence, clusters such as Zintl ions usually retain their structure upon isoelectronic replacement of some or all atoms. Here, a deviation from this common observation is presented, namely the formation of (Sn_5_Sb_3_)^3−^ (**1**), a rare example of an eight‐vertex Zintl ion, and an unprecedented example of a Zintl ion synthesized by solution means that has an arachno‐type structure according to the Wade–Mingos rules. Three structure‐types of interest for (Sn_5_Sb_3_)^3−^ were identified by DFT calculations: one that matched the X‐ray diffraction data, and two that that were reminiscent of fragments of known clusters. A study on the isoelectronic series of clusters, (Sn_x_Sb_8−x_)^2−x^ (x=0–8), showed that the relative energies of these three isomers vary significantly with composition (independent of electron count) and that each is the global minimum at least once within the series.

## Graphical abstract

**An eight‐vertex Zintl anion** with an *arachno*‐type structure obtained from solution, (Sn_5_Sb_3_)^3−^ (**1**), was synthesized as its [^*n*^Bu_4_P]^+^ salt. Calculations on the isoelectronic series (Sn_*x*_Sb_8−*x*_)^2−*x*^ (*x*=0–8) were performed to investigate possible alternative structures of **1**, which revealed that the preferred structures of the isomers vary significantly with composition, independent of electron count.

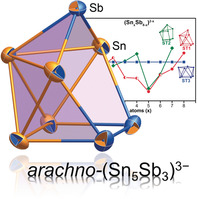

The investigation of metal clusters provides a window into the initial steps in the formation of metal nanoparticles or even bulk metal in general. Even though the molecular scale does not inform exactly about the metallic state, the formation of first bonds and the structures of seed clusters play a crucial role for the subsequent growth of a particle. As such, the isolation and thorough analysis of different types of metal clusters has been a very active field over the years.^1^ One particularly active field of pure metal clusters is that of homometallic or polymetallic anions composed of main‐group elements, so‐called Zintl clusters, which allow for an extremely broad range of compositions and structures owing to the numerous possible elemental combinations.^2^

Classical Zintl ion chemistry has long borrowed the Wade–Mingos rules to relate structures to the number of bonding or valence electrons.^3^ These rules were developed to explain the observed structures of borane clusters,^4^ and are applicable to Zintl ions owing to the isoelectronic relationship between fragments {B‐H} and E atoms (E=Si–Pb), which both contribute two electrons to cluster bonding orbitals. In this construct, the clusters are classified according to the number of “missing” vertices relative to a parent deltahedron; the labels of *closo*, *nido*, *arachno*, and *hypho* are given to clusters with 0, 1, 2, and 3 missing vertices and cluster skeletal electron (SE) counts of 2*n*+2, 2*n*+4, 2*n*+6, and 2*n*+8, where *n* is the number of actual cluster vertices. Thus far, deltahedral Zintl ions with 4–7, 9, 10, and 12 vertices have been crystallized and structurally characterized as their respective salts from ethylenediamine (en) or liquid ammonia solutions (Figure 1 a).

**Figure 1:**
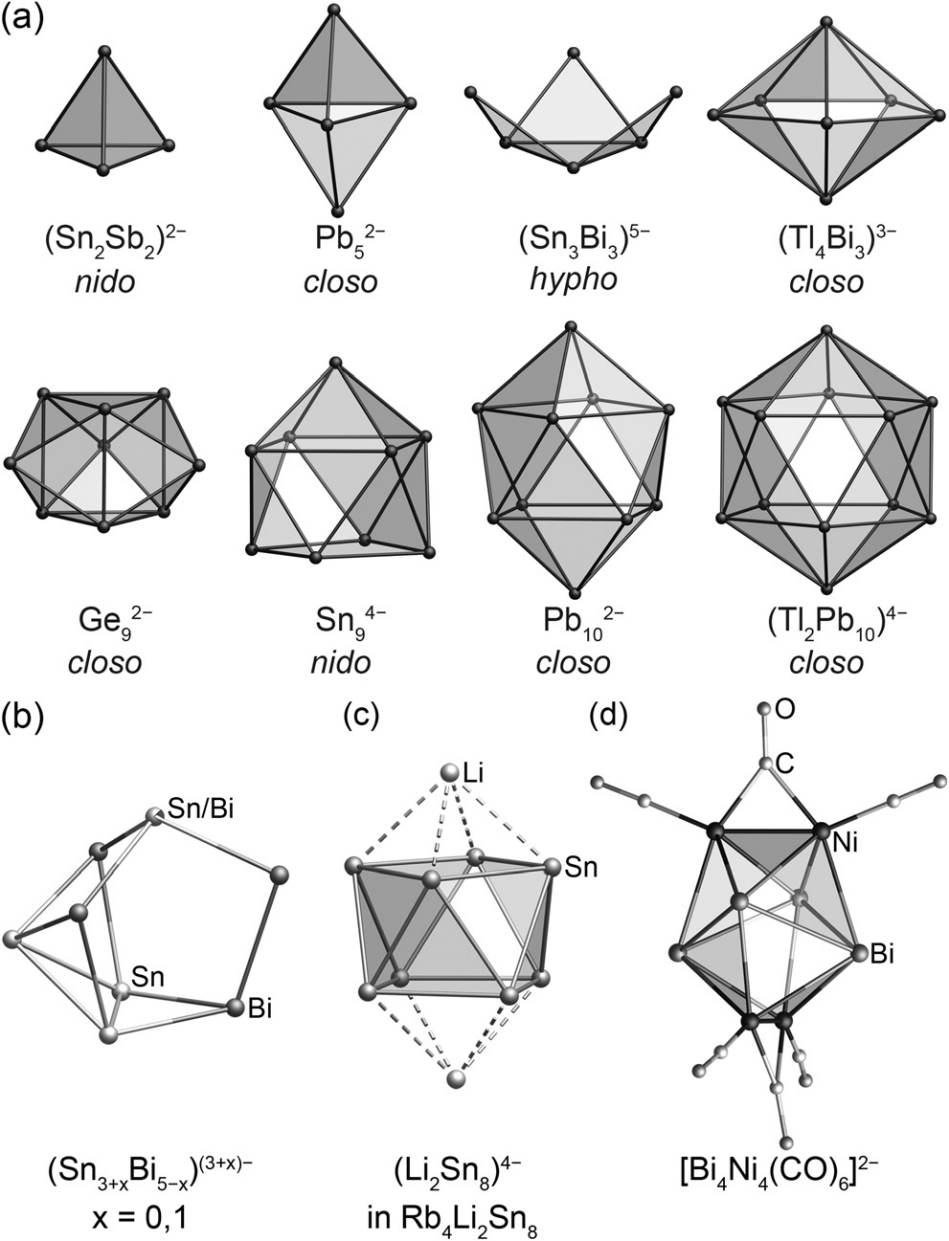
a) Representative structures of Zintl ions comprising elements of Groups 14 and 15 that have deltahedral or deltahedra‐derived structures, which have been synthesized by solution means.^5^ b) The structure of the non‐deltahedral isostructural anions, (Sn_3_Bi_5_)^3−^ and (Sn_4_Bi_4_)^4−^.5c, 6 c) The (Li_2_Sn_8_)^4−^ subunit of Rb_4_Li_2_Sn_8_ which comprises an *arachno*‐Sn_8_^4−^ moiety.^7^ d) The *closo*‐type heterometallic cluster, [Bi_4_Ni_4_(CO)_6_]^2−^.^8^

Most of Zintl clusters isolated by solution means are of the *closo*‐ or *nido*‐type. The only example of a *hypho*‐cluster is (Sn_3_Bi_3_)^5−^, which was synthesized in liquid ammonia and its high charge density compensated for by close contacts with Rb^+^ cations.5c Of particular note is the lack of deltahedral Zintl ions synthesized by solution means that have either 8 vertices or that are classified as *arachno*.

There are published examples of non‐deltahedral Zintl ions, clusters within Zintl phases obtained by solid state approaches, and heterometallic anions with 8 atoms. The non‐deltahedral anions (Sn_3_Bi_5_)^3−^ and (Sn_4_Bi_4_)^4−^ were synthesized by extraction of “RbSnBi” in liquid ammonia.5c, 6 The two isostructural and isoelectronic anions have non‐deltahedral nortricyclane‐like structures, typical of Pn_7_^3−^ (Pn=P, As, Sb, Bi), but they are bimetallic (Sn/Bi) and exhibit an additional tin atom capping one side (Figure 1 b). They have 40 valence electrons (VEs), the amount expected for a molecule comprising only pnictogen atoms. The A_4_Li_2_Sn_8_ (A=K, Rb) Zintl phases contain {Sn_8_^6−^} subunits with square antiprism structures, which are capped on either side by Li^+^ cations (Figure 1 c).^7^ These subunits are *arachno*‐type, with 38 VEs, thus 22 SEs (2*n*+6, *n*=8), and two missing vertices relative to the parent deltahedron (discounting the Li^+^ cations). A hypothetical *closo*‐type Zintl cluster with 8 atoms would have 18 SEs and adopt a trigonal dodecahedral structure. While there are no such homoatomic examples, the heterometallic cluster [Bi_4_Ni_4_(CO)_6_]^2−^ was shown to have this structure, and indeed comprises 18 SEs (12 from the bismuth atoms, 4 from the {Ni_2_(CO)_3_} fragments, and 2 from the charge).

As indicated by the examples given in Figure 1, the prediction of isostructural molecules by the pseudo‐element concept has proved to be very robust for molecular clusters to date, with the only exceptions being found for some intermetalloid clusters (owing to the active involvement of s and d electrons of inner endohedral transition metal atoms),^9^ or in the case of odd electron numbers that led to (explainable) distortions.^10^

However, our results reveal that structural trends within isoelectronic anions of Zintl compounds can be influenced predominantly by the actual composition, independent of electron count. In this work, we present the X‐ray crystallographic and spectroscopic characterization of *arachno*‐(Sn_5_Sb_3_)^3−^ (**1**), which is rare example of an 8‐vertex Zintl ion. A quantum chemical investigation confirmed the heretofore unknown structure of **1** to be the global minimum. The study identified two additional local minimum structure types of higher total energies that correlate to 8‐atom fragments in known, larger intermetalloid clusters. The different architectures of species with the same composition in different chemical environments prompted us to perform an expanded quantum chemical investigation of the isoelectronic series (Sn_*x*_Sb_8−*x*_)^2−*x*^. It revealed that the energies of these three structure types relative to each other vary greatly with stoichiometry, and that each structure type is the global minimum at least once in the series. Hence, the strict connection of valence electron count of main‐group metal clusters with their structure seems to require refinement.

The binary Zintl anion **1** was isolated as a component of the highly air and moisture sensitive salt [^*n*^Bu_4_P]_3_ **1**, which crystallized in low yield from a concentrated mixture of [^*n*^Bu_4_P]Br and K_2_SnSb in en (see the Supporting Information for details). Analysis by single crystal X‐ray diffraction (SCXRD) revealed a salt comprising three tetrabutylphosphonium cations and an anion comprising eight tin or antimony atoms that formed a cluster with seven 3‐atom faces and adjacent 4‐ and 5‐atom faces (Figure 2 a). This structure is derived from that of a 10‐vertex deltahedron by removal of two non‐adjacent 4‐ and 5‐connected vertices, and is thus *arachno* according to the Wade–Mingos rules (Figure 3 b, left). Tin and antimony cannot be distinguished by SCXRD; however, analysis of the same crystal by micro‐X‐ray fluorescence spectroscopy (μ‐XFS) supported an approximate Sn:Sb ratio of 5:3 (Supporting Information, Figure S4 and Table S2). A formulation of (Sn_5_Sb_3_)^3−^ for the anion was further supported by the number of SEs (2*n*+6=22, *n*=8) matching that of an *arachno* classification, and thus the observed structure.

**Figure 2:**
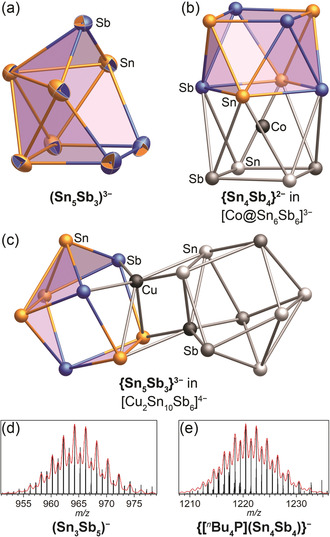
a) The structure of the anion (Sn_5_Sb_3_)^3−^ (**1**);^17^ the two‐color ellipsoids (50 % probability) indicate mixed occupation with the outer color representing the occupation in the global minimum isomer that was obtained upon geometry optimization by quantum chemical calculations with DFT methods (see text). b),c) 8‐atom fragments (highlighted in color) within the reported anions [Co@Sn_6_Sb_6_]^3−^ (b),^11^ and [{CuSn_5_Sb_3_}_2_]^4−^ (c).^12^ Sn orange, Sb blue. d),e) Mass peaks of (Sn_3_Sb_5_)^−^ (d) and {[^n^Bu_4_P](Sn_4_Sb_4_)}^−^ (e), obtained from a 2:1 solution of [^*n*^Bu_4_P]Br and K_2_SnSb in en under electrospray ionization mass spectrometry (ESI‐MS) conditions; red lines represent the calculated mass envelopes.

**Figure 3:**
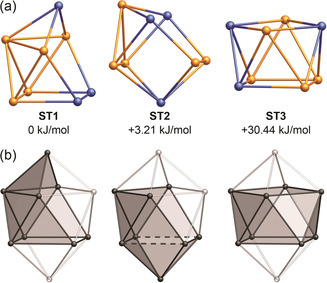
a) The three lowest‐energy geometry‐optimized isomeric minimum structures for (Sn_5_Sb_3_)^3−^ identified by genetic algorithm (GA) paired with reassignment of atomic positions (RP) by first‐order perturbation theory in the nuclear charge. b) A 10‐vertex, *closo*‐type deltahedral structure (*D*_4*d*_) with shading showing the relationships to the above structural motifs for (Sn_5_Sb_3_)^3−^. The dashed lines represent broken contacts in the derivation of ST2 from the parent deltahedron.

Attempts at characterization of the crystals by mass spectrometry did not produce a parent peak for (Sn_5_Sb_3_)^3−^, but rather a mixture of anions of lower charge. Mass spectral characterization of a 2:1 mixture of [^*n*^Bu_4_P]Br and K_2_SnSb in en revealed the presence of (Sn_3_Sb_5_)^1−^ and (Sn_4_Sb_4_)^2−^, both of which are isoelectronic with **1**, in addition to (Sn_2_Sb_2_)^2−^ and (Sn_6_Sb_3_)^1−^ (Figure 2 d,e; see the Supporting Information for details).

To further corroborate the evidence for a (Sn_5_Sb_3_)^3−^ composition for **1**, as well as to determine its global minimum structure, we conducted a quantum chemical study using a genetic algorithm (GA) extended by reassignment of atom‐type positions by first‐order perturbation theory in the nuclear charge (RP), denominated as GA‐RP in this combination (see methods section for details).^13^ We conducted this study on the three most reasonable candidates with closed‐shell configurations: (Sn_7_Sb_1_)^3−^, (Sn_5_Sb_3_)^3−^, and (Sn_3_Sb_5_)^3−^. The global minima identified for (Sn_7_Sb_1_)^3−^ and (Sn_3_Sb_5_)^3−^ resemble distorted deltahedra and capped nortricyclane structures, respectively (Supporting Information, Figure S8). The latter structure is known from the bismuth analogue, (Sn_3_Bi_5_)^3−^.5c As expected, the GA‐RP procedure identified a global minimum structure type (ST) for (Sn_5_Sb_3_)^3−^ that closely matched the crystallographic data (ST1; Figure 3 a, left; see the Supporting Information, Figure S9 and Table S3 for a comparison of bond lengths). Furthermore, the GA‐RP determined that the three antimony atoms in the global minimum isomer of (Sn_5_Sb_3_)^3−^ occupy the positions with contacts to only three other atoms, as expected for pnictogens. Thus, the quantum chemical calculations, crystallographic data, and X‐ray fluorescence spectroscopic data all support a formulation of [^*n*^Bu_4_P]_3_(Sn_5_Sb_3_) for the isolated red crystals.

Further to the global minimum of **1**, the GA‐RP study also identified two other STs as local minima. The second most stable ST according to the GA‐RP analysis (ST2, +3.2 kJ mol^−1^), comprises five 3‐atom and two 4‐atom faces, as well as one 5‐atom face (Figure 3 a, middle). This structure is derived from the parent 10‐vertex deltahedron by removal of two adjacent vertices followed by the elongation of two opposite contacts to yield two (near) square faces (Figure 3 b, middle; elongation denoted by dashed lines in the bottom part). A concomitant slight shift in adjacent atom positions leads to the nearly flat 5‐atom ring in ST2. The third most stable ST identified by the GA‐RP (ST3, +30.4 kJ mol^−1^) was a square antiprism (Figure 3 a, right). The structure of ST3 is also derived from a parent 10‐vertex deltahedron, but with opposite 4‐connected vertices removed (Figure 3 b, right). Overall, the situation is reminiscent of the arachno/*iso*‐arachno relationship known from boranes.

No 8‐vertex main group‐element clusters of the type ST2 or ST3 have been isolated; however, there are known coordination compounds and intermetalloid clusters with (formally) isoelectronic and structurally related moieties. Square anti‐prism motifs (ST3) are found in a number of related compounds, such as {Tl_2_Bi_6_}^2−^ in [Tl_2_Bi_6_{Ru(cod)}]^2−^,^14^ and {Bi_8_}^2+^ in (CuBi_8_)[AlCl_4_]_3_,^15^ {Sn_8_}^6−^ in Rb_4_Li_2_Sn_8_,^7^ and {Sn_4_Sb_4_}^2−^ in [Co@Sn_6_Sb_6_]^3−^ (Figure 2 b).^11^ The structural motif of ST2 is less common. The heterometallic cluster [Sb_6_(RuCp*)_2_]^2−^ has a remarkably similar 8‐vertex core.^16^ Indeed, taking the {RuCp*}^−^ moieties as 2−e^−^ donors leads to the expected SE count of 22. Most notably, however, the global minimum isomer of the heterometallic dimer, [Cu_2_Sn_10_Sb_6_]^4−^ (Figure 2 c),^12^ comprises a {Sn_5_Sb_3_}^3−^ fragment nearly identical to ST2 (Figure 3 a, middle). Thus it is plausible that this isomer of **1** is the active species in the formation of [Cu_2_Sn_10_Sb_6_]^4−^, while it crystalizes in its minimum structure ST1.

In contrast to the above‐mentioned compounds, we know of no corresponding examples of 8‐vertex cluster moieties that resemble **1** (ST1). Instead, the square anti‐prism (ST3) is the most commonly observed motif for such moieties with 22 SEs, but this structure type is significantly less stable than ST1 for the (Sn_5_Sb_3_)^3−^ anion (30.4 kJ mol^−1^). We suspected that the relative stability of the ST1 structure type was related to its heteroatomic composition. We therefore carried out a comprehensive GA‐RP investigation to determine the relative energies of ST1, ST2, and ST3 for a series of molecules, (Sn_*x*_Sb_8−*x*_)^2−*x*^ (*x*=0–8), all of which are isoelectronic with **1**.

Figure 4 shows a plot of the energies of the most stable isomers of each structure type for the series (Sn_*x*_Sb_8−*x*_)^2−*x*^ (*x*=0–8; see the Supporting Information for more detail). These energies were plotted relative to ST3, as this structure type was both present and undistorted for all eight stoichiometries. ST1 was also identified for all stoichiometries; however, in four instances the most stable isomer was distorted from the ideal structure (*x*=2, 3, 4, 6; indicated by half‐filled circles in Figure 4). Without implementing symmetry restrictions, we only found examples of ST2 for *x*=2–7. All instances of ST2 that we identified contained a mirror plane, whereas ST1 and ST3 did not appear to have any symmetry restrictions. We note that ST2 can be not be converted to ST1 under retention of the topological relationship of all atoms.

**Figure 4:**
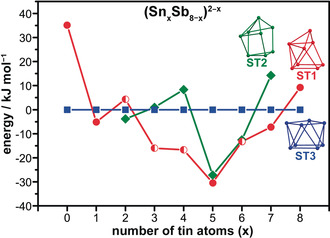
Comparison of the energies of the local minima of the three structure types shown in Figure 3 a, for (Sn_*x*_Sb_8−*x*_)^2−*x*^ (*x*=0–8), plotted relative to the energy of ST3. Distortions of ST1 from the ideal structure (observed for *x*=2, 3, 4, 6) are indicated by half‐filled circles. See the Supporting Information, Table S4 for the molecular structures.

Figure 4 demonstrates that there is a heteroatomic effect on the structure of (Sn_*x*_Sb_8−*x*_)^2−*x*^, independent of the number of valence electrons (in this case 38). There are three important features of these results: 1) each of the three structure types is the most stable in at least one instance, 2) ST3 is only the most stable structure type for the homoatomic ions (Sn_8_^6−^ and Sb_8_^2+^), and 3) the energies of the most stable versions of ST1 converge on the stoichiometry of **1**. Therefore, while all three isomers are *arachno*‐type structures, the structure that presents for (Sn_*x*_Sb_8−*x*_)^2−*x*^ (that is, which two vertices are lacking) is a function of composition. In ST3, all atom positions are equivalent, possibly explaining its favorability for the homoatomic compositions (Sb_8_^2+^ and Sn_8_^6−^). In contrast, ST1 has five unique atomic environments, which explains why heteroatomic effects can override the valence electron count under certain circumstances. These findings add a hitherto undefined dimension to the relationship between structure and composition for Zintl ions. We note in passing that we also tested other electron counts (32, 34, 36, and 40) by the same method, the results of which are not detailed here, as they exceed the scope of this work. However, it is briefly stated here that our findings are consistent in the sense that there is no single global minimum structure across these isoelectronic series.

In conclusion, we report the synthesis and characterization of a phosphonium salt of the first *arachno*‐type Zintl anion obtained from solution synthesis, which is also a rare example of such a cluster having 8‐vertices. Quantum chemical considerations of all potential compositions (Sn_*x*_Sb_8−*x*_)^2−*x*^ (*x*=0–8) not only supported the composition of **1** to be (Sn_5_Sb_3_)^3−^, but also indicated that isoelectronic anions in this case do not prefer the same architectures, but rather exhibit different minimum structures. This is a new facet of the pseudo‐element concept, which usually suggests equivalent structures for isoelectronic molecules.

## Conflict of interest

The authors declare no conflict of interest.

## Supporting information

As a service to our authors and readers, this journal provides supporting information supplied by the authors. Such materials are peer reviewed and may be re‐organized for online delivery, but are not copy‐edited or typeset. Technical support issues arising from supporting information (other than missing files) should be addressed to the authors.

Supplementary
