# Supplementary material for: The Arachno‐Zintl Ion (Sn5Sb3)3− and the Effects of Element Composition on the Structures of Isoelectronic Clusters: Another Facet of the Pseudo‐Element Concept
Source: Angew Chem Int Ed Engl. 2020 Jul 8;59(34):14251–5. doi: 10.1002/anie.202002863 (PMC7496391; doi:10.1002/anie.202002863)
Supplement: Supplementary file 1 — Supplementary [file ANIE-59-14251-s001.pdf]

Angewandte Chemie
Eine Zeitschrift der Gesellschaft Deutscher Chemiker

Supporting Information

The *Arachno*-Zintl Ion $(Sn_5Sb_3)^{3-}$ and the Effects of Element Composition on the Structures of Isoelectronic Clusters: Another Facet of the Pseudo-Element Concept

*Robert J. Wilson, Florian Weigend, and Stefanie Dehnen**

anie_202002863_sm_miscellaneous_information.pdf

Synthesis methods

General: All reactions were performed in an argon atmosphere using Schlenk techniques or a glovebox. Ethane-1,2-diamine (en) (Aldrich, 99.8%) was distilled from CaH_2 and stored over 3 Å molecular sieves. Toluene (Acros Organics, 99%) was distilled from sodium-potassium alloy and stored over 3 Å molecular sieves. $[^{n}Bu_4P]Br$ was dried under vacuum overnight. K_2SnSb was prepared by stoichiometric fusion of the elements at 950°C for 48 hours in a niobium tube, sealed within an evacuated silica ampule.

Synthesis of $[^{n}Bu_4P]_3(Sn_5Sb_3)$ ($[^{n}Bu_4P]_3$1): K_2SnSb (100 mg, 0.31 mmol) and $[^{n}Bu_4P]Br$ (213 mg, 0.63 mmol) were combined in a Schlenk tube and dissolved in 5 ml of en. After stirring for 24 h at room temperature, the dark red-brown solution was filtered through a 0.45 μm PTFE syringe filter into a 25 mL Schlenk flask. The sample was subsequently reduced in volume under vacuum to ~1 mL and stored at 5 °C. After one week, small red, block-like crystals were present in the bottom of the flask. A crystal suitable for X-ray diffraction was picked quickly under flowing argon. Approximately 3-5 mg of highly air and moisture sensitive crystals were subsequently washed once with 2 mL of toluene and isolated from the flask. A combination of single crystal X-ray diffraction, X-ray fluorescence spectroscopy, and quantum chemical calculations identified the product as $[^{n}Bu_4P]_3(Sn_5Sb_3)$. The crystallization of $[^{n}Bu_4P]_3$**1** stands in contrast to the formation of $[K(crypt\text{-}222)]_2(Sn_2Sb_2)\cdot en$, which crystallizes from en extractions of K_2SnSb and crypt-222. The formation of **1** rather than $(Sn_2Sb_2)^{2-}$ is likely attributable to either cation packing effects, oxidation of the Zintl anions, or both.

Crystallographic data for $[^{n}Bu_4P]_3(Sn_5Sb_3)$ ($[^{n}Bu_4P]_3$**1**)

The data set for $[^{n}Bu_4P]_3(Sn_5Sb_3)$ was collected on a Bruker D8 Quest with a CMOS detector. Structural data was collected at T = 100(2) K with $Mo_{K\alpha}$ radiation (λ = 0.71073 Å). The structure was solved by direct methods and refined by full-matrix-least-squares methods against F^2 with SHELXL-2014[1] and Olex2.[2]. General crystallographic data are listed in Table S1. Sb, Sn, P, and C atoms were refined using anisotropic displacement parameters. Hydrogen atoms were placed in idealized positions and their displacement parameters were fixed to be 20% larger than those of the attached carbon atoms. Figure S1 and Figure S2 show the crystal packing and the asymmetric unit, respectively, of $[^{n}Bu_4P]_3(Sn_5Sb_3)$. The highest peak and the deepest hole of the final difference Fourier map (5.23 $e^{-}\cdot Å^{-3}$/–1.29 $e^{-}\cdot Å^{-3}$) were located 0.87 Å from Sb1 and 0.47 Å from Sn1 (see the numbering scheme in Figure S3).

Table S1. Crystallographic data, structure solution and refinement details of $[^{n}Bu_4P]_3(Sn_5Sb_3)$ ($[^{n}Bu_4P]_3$**1**).

**Compound**	$[^{n}Bu_4P]_3$**1**
empirical formula	$C_{48}H_{108}P_3Sb_3Sn_5$
formula weight [g mol^{-1}]	1736.95
temperature [K]	100
crystal color, shape	dark red, block
crystal size [mm^3]	1.98 x 10^{-3}
crystal system	monoclinic
space group	$P2_1/n$
a [Å]	11.6862(7)
b [Å]	24.5394(13)
c [Å]	23.6909(13)
α [°]	90
β [°]	102.580(2)
γ [°]	90
V [Å^3]	6630.8(6)
Z, ρ_{calc} [g cm^{-3}]	4
μ [mm^{-1}]	3.153
absorption correction type	multi-scan
2θ range [°]	4.62 to 52.74
reflections collected	105205
ind. reflections / R_{int} / R_{sigma}	13549 / 0.0751 / 0.0448
restraints/parameters	204/588
Final R indexes [$I \geq 2\sigma(I)$]	R1 = 0.0697, wR2 = 0.1810
Final R indexes [all data]	R1 = 0.0852, wR2 = 0.1918
goodness-of-fit on F^2	1.07
max peak/hole [e Å^{-3}]	5.23/−1.29
CCDC number	1981397

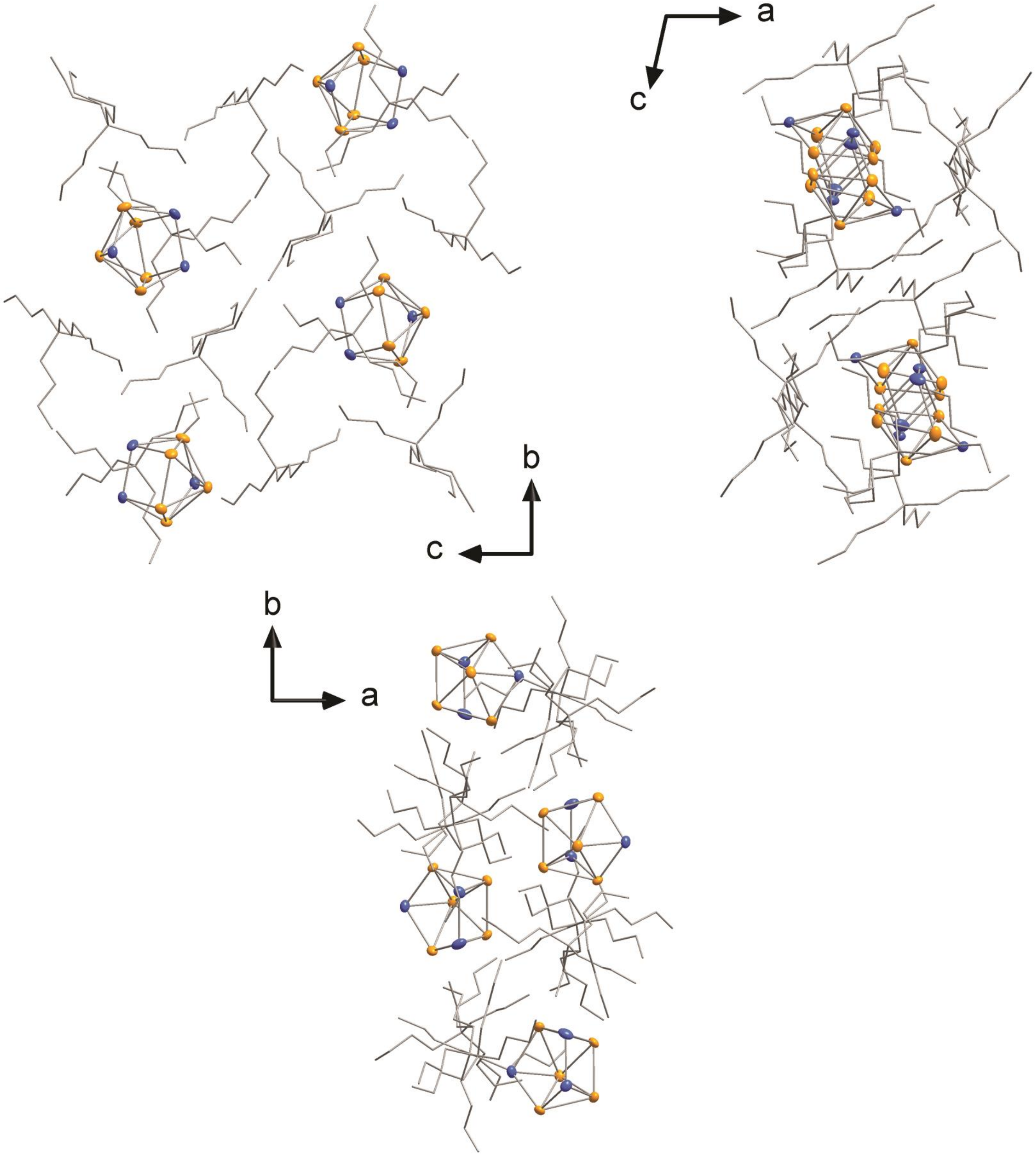

Figure S1. Packing of cations and anions in $[^nBu_4P]_3(Sn_5Sb_3)$ ($[^nBu_4P]_3$**1**) viewed down the a (top left), b (top right), and c (bottom) axes. Displacement ellipsoids are drawn at 50% probability. Elements are represented by the following colors: Sn, orange; Sb, blue. The indicated positions of tin and antimony correspond to the calculated global minimum isomer that was obtained upon geometry optimization by quantum chemical calculations with DFT methods (see main text). Tetrabutylphosphonium cations drawn as gray sticks and hydrogen atoms have been omitted for clarity.

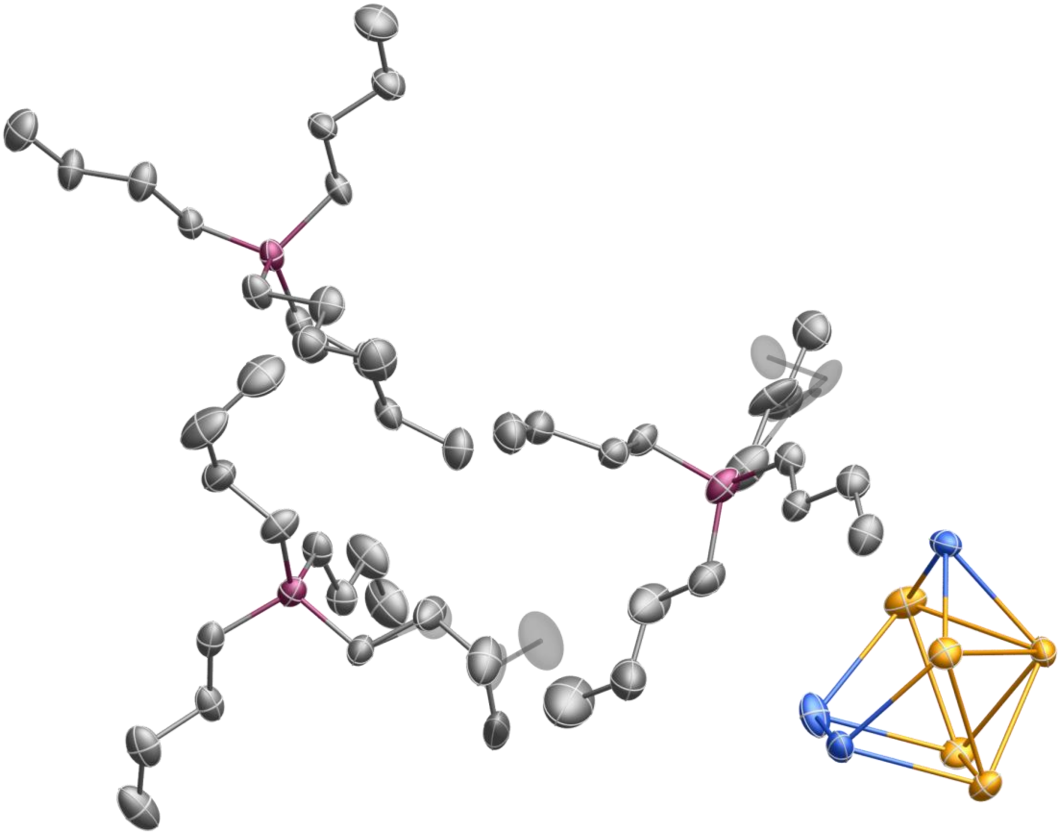

Figure S2. Asymmetric unit of $[^n\text{Bu}_4\text{P}]_3(\text{Sn}_5\text{Sb}_3)$ ($[^n\text{Bu}_4\text{P}]_3$**1**). Thermal ellipsoids are drawn at 50% probability. Elements are represented by the following colors: Sn, orange; Sb, blue; P, purple; C, grey. The indicated positions of tin and antimony correspond to the calculated global minimum isomer that was obtained upon geometry optimization by quantum chemical calculations with DFT methods (see main text). Positions of disordered atoms are given in semi-transparent mode. Hydrogen atoms have been omitted for clarity.

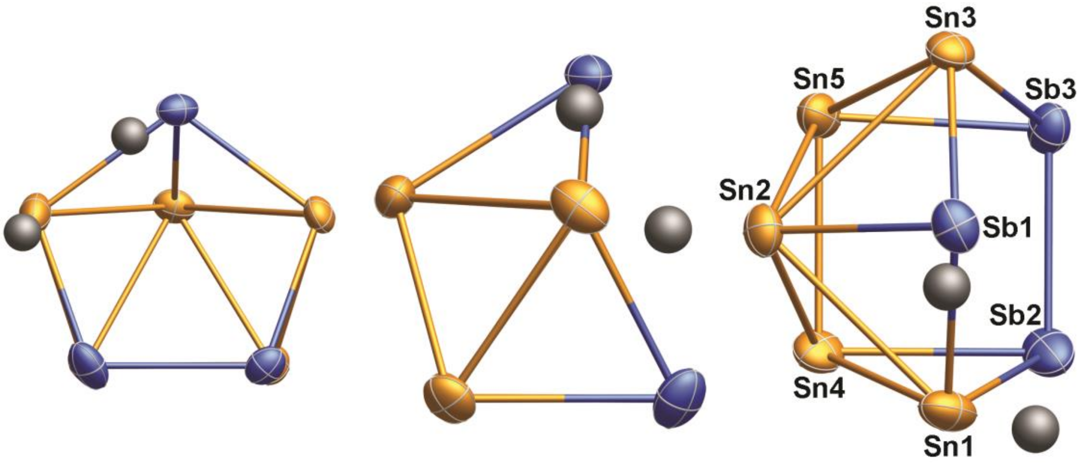

Figure S3. Three views of the binary anion in the crystal structure of $[^n\text{Bu}_4\text{P}]_3(\text{Sn}_5\text{Sb}_3)$ ($[^n\text{Bu}_4\text{P}]_3$**1**), shown along with the two highest Q peaks of electron density on the difference Fourier map. Displacement ellipsoids (Sn, Sb) are drawn at 50% probability. The indicated positions of tin and antimony correspond to the calculated global minimum isomer that was obtained upon geometry optimization by quantum chemical calculations with DFT methods (see main text). Elements are represented by the following colors: Sn, orange; Sb, blue; Q, grey. The figures clearly indicate that the highest electron density does not correlate with the reasonable position of a ninth or tenth metal atom to form a *nido* or *closo* cluster.

Micro-X-Ray Fluorescence Spectroscopy (µ-XFS)

All µ-XFS measurements were performed with a Bruker M4 Tornado, equipped with a Rh-target X-ray tube and a silicon drift detector. The emitted fluorescence photons were detected with an acquisition time of 100 s. Quantification of the elements is achieved through deconvolution of the spectra. Results are summarized in Table S2. Rhodium is omitted from the quantification results. Figure S4 shows the spectrum for a single crystal of $[^{n}Bu_4P]_3(Sn_5Sb_3)$ ($[^{n}Bu_4P]_3$**1**) along with the results of the deconvolution algorithm.

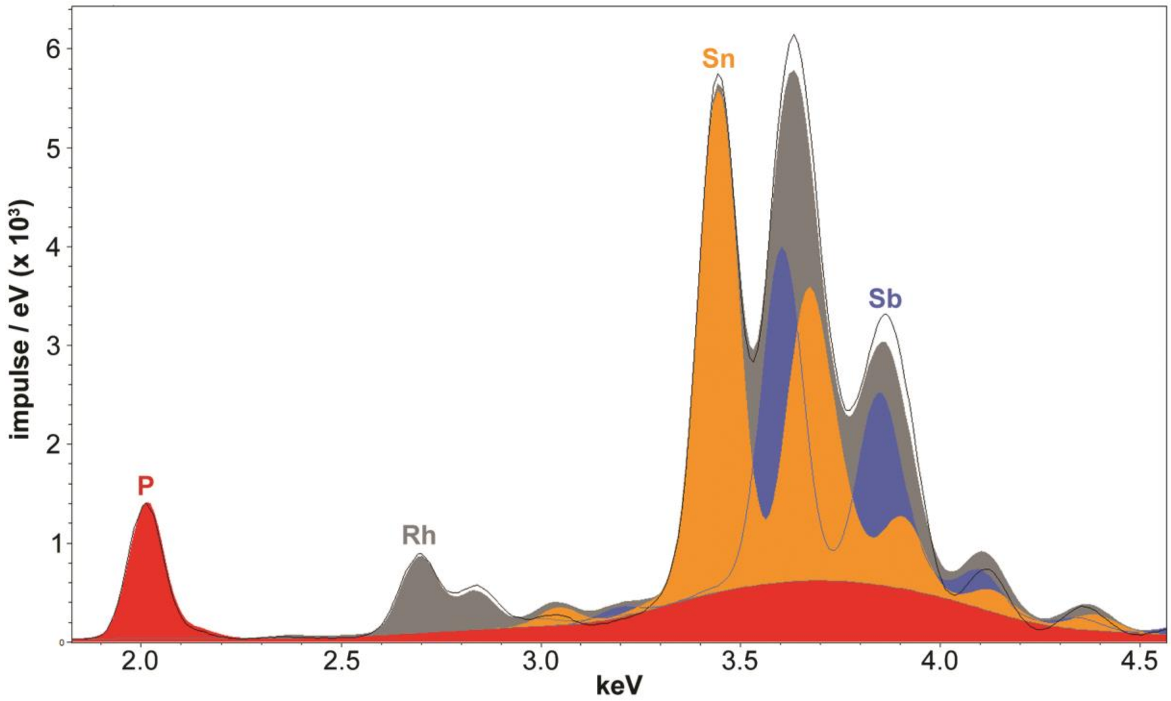

Figure S4. µ-XF spectrum of $[^{n}Bu_4P]_3(Sn_5Sb_3)$ ($[^{n}Bu_4P]_3$**1**; black line) with the results of the deconvolution algorithm (solid, dark grey). Element colors are as follows: P (red), Sn (orange), Sb (blue), Rh (light gray).

Table S2. Results of semi-quantitative analysis of µ-XF spectrum for a single crystal of $[^{n}Bu_4P]_3(Sn_5Sb_3)$ ($[^{n}Bu_4P]_3$**1**).

	Experimental wt. %	Calculated wt. %	Experimental atom %	Calculated atom %
P – K	8.57	8.78	26.62	27.27
Sn – L	57.78	56.10	46.81	45.45
Sb – L	33.65	34.52	26.58	27.27

Mass Spectrometry

The mass spectrum was recorded on the reaction mixture (2[^{n}Bu$_4$P]Br + K_2SnSb in en), from which crystals of [^{n}Bu$_4$P]$_3$**1** could be obtained (see synthesis methods), with a Thermo Fischer Scientific Finnigan LTQ-FT spectrometer in the negative ion mode. The sample was prepared inside of a glovebox, where the materials were dissolved in anhydrous en, stirred overnight, and then filtered through a Teflon syringe filter with a pore size of 0.45 μm. The solution was injected into the spectrometer with a gastight 250 μL Hamilton syringe by syringe pump infusion. All capillaries within the system were washed with dry en for 30 minutes before the measurement to avoid decomposition reactions.

The following ESI parameters were used: spray voltage 3.6 kV, capillary temp 300 °C, capillary voltage –12, tube lens voltage –86.7, sheath gas 45, sweep gas 0, auxiliary gas 40.

Figure S5 shows the overview spectrum, high-resolution peaks are provided in Figures S6 and S7.

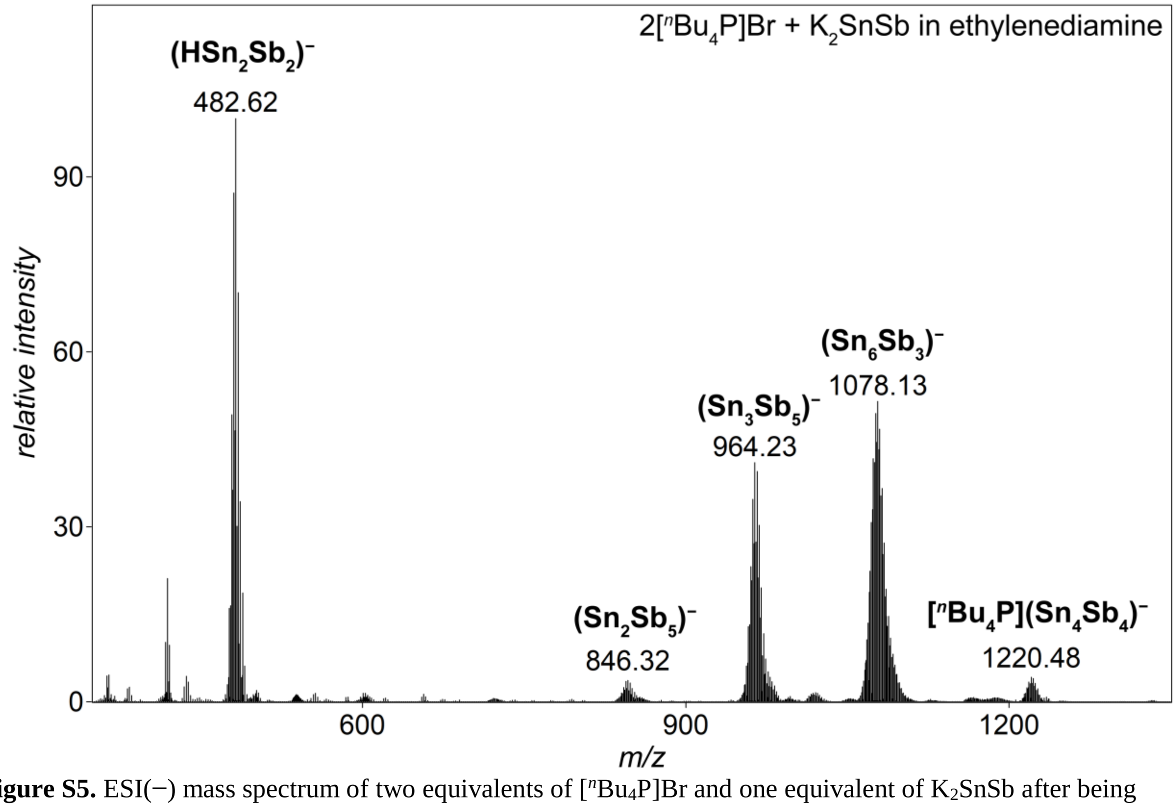

Figure S5. ESI(−) mass spectrum of two equivalents of [^{n}Bu$_4$P]Br and one equivalent of K_2SnSb after being dissolved in en, stirred overnight, and filtered through a 0.45 μm PTFE filter.

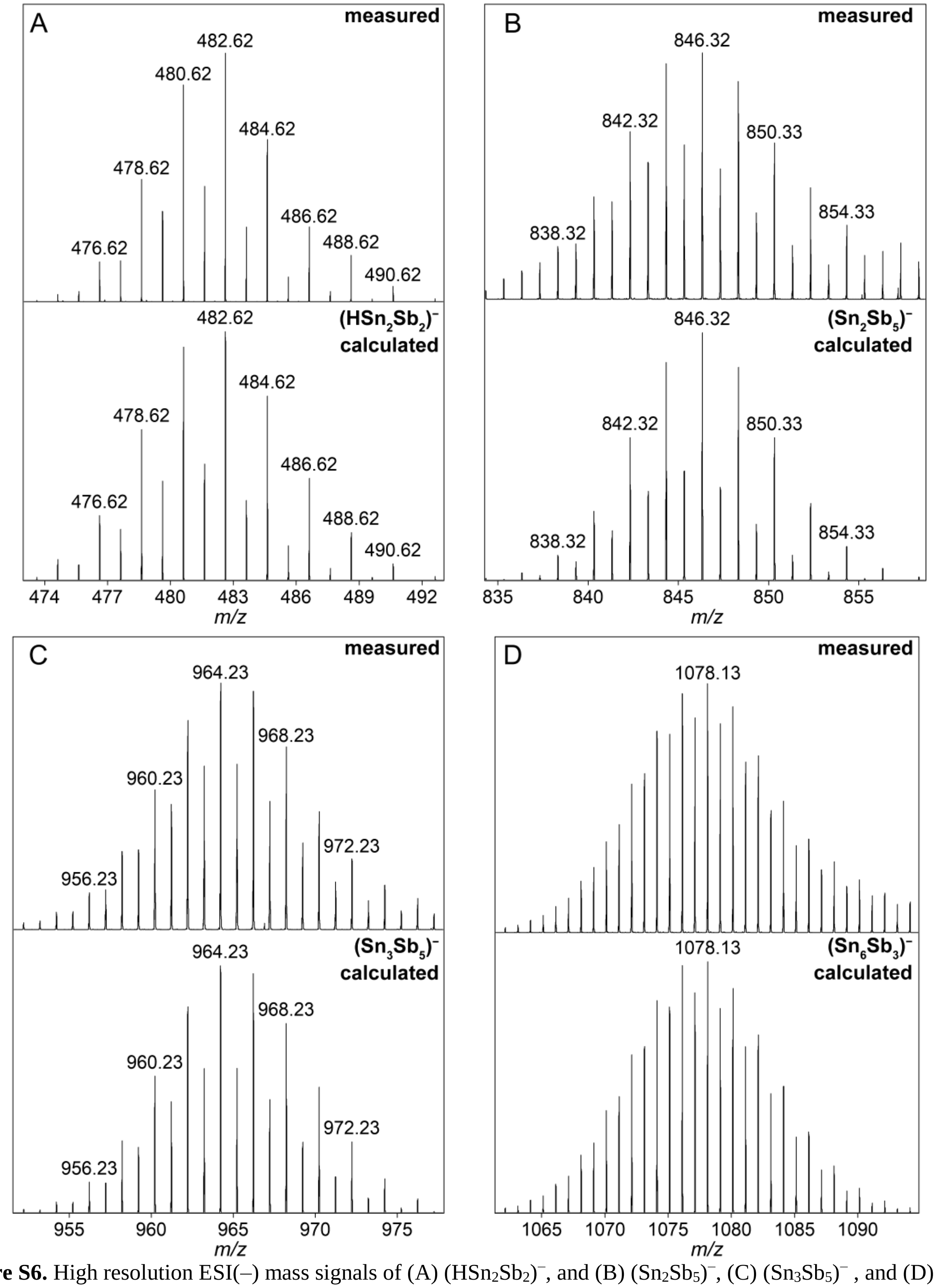

Figure S6. High resolution ESI(–) mass signals of (A) $(HSn_2Sb_2)^-$, and (B) $(Sn_2Sb_5)^-$, (C) $(Sn_3Sb_5)^-$, and (D) $(Sn_6Sb_3)^-$.

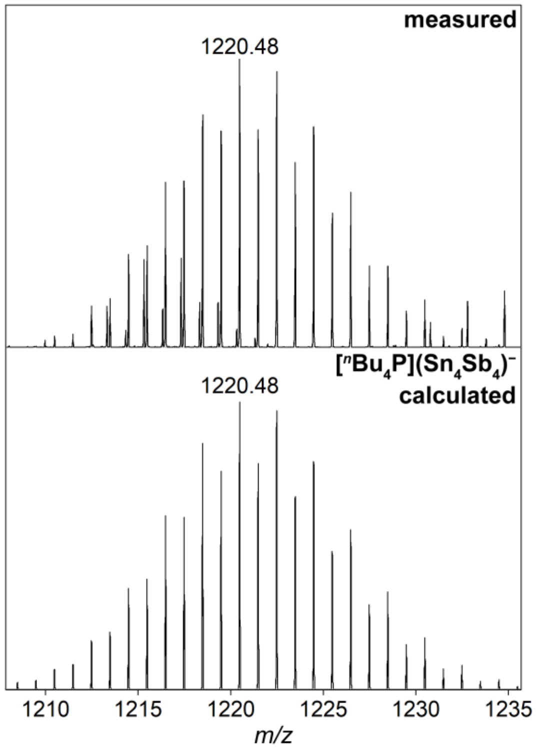

Figure S7. High resolution ESI(–) mass signal $[^{n}Bu_4P](Sn_4Sb_4)^{-}$.

Quantum Chemical Calculations

Calculations were carried out with TURBOMOLE.[2] For Sn and Sb atoms, an ECP-28[4] was used together with a triple zeta basis (def2-TZVP).[5] The conductor-like screening model (COSMO)[6] was applied for charge compensation with the default parameters. Structures were optimized using the TPSS[7] functional and medium sized grids (gridsize m3) together with a self-consistent field (SCF) convergence threshold of 10^{-7} Eh. The resolution of the identity approximation (RI-J) was applied with the corresponding auxiliary basis sets.[8]

Figure S8 shows the calculated global minimum isomers of $(Sn_7Sb_1)^{3-}$, $(Sn_5Sb_3)^{3-}$, and $(Sn_3Sb_5)^{3-}$, demonstrating that $(Sn_5Sb_3)^{3-}$ best matches the X-ray structure of the anion shown in Figure 2a. A comparison of the experimental and the calculated structures of the $(Sn_5Sb_3)^{3-}$ anion (**1**) are shown in Figure S9. Corresponding bond lengths are provided in Table S3. Calculated structures and corresponding energies of the isomers of $(Sn_xSb_{8-x})^{2-x}$ are given in Table S4. The methods are detailed in the main document.

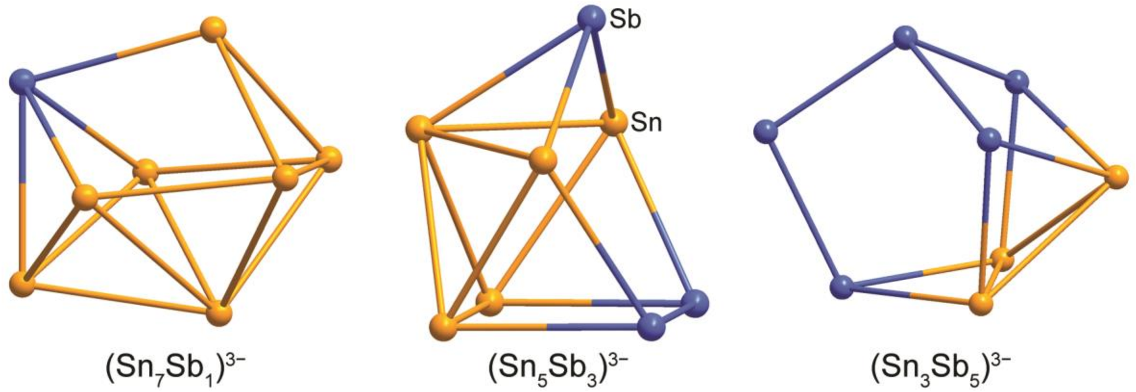

Figure S8. Lowest energy calculated isomers of $(Sn_7Sb_1)^{3-}$, $(Sn_5Sb_3)^{3-}$, and $(Sn_3Sb_5)^{3-}$.

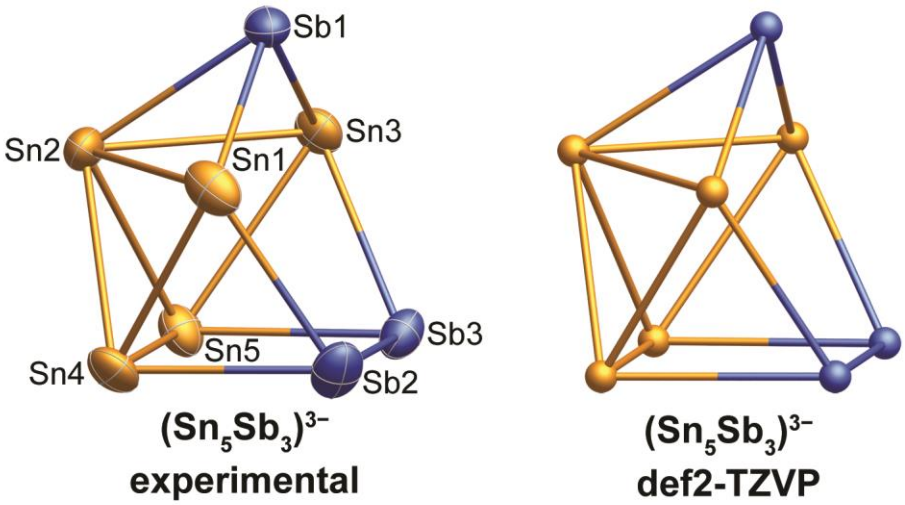

Figure S9. Comparison of experimental and calculated structures of $(Sn_5Sb_3)^{3-}$. See Table S3 for structural data.

Table S3. Comparison of bond lengths [Å] and selected angles [°]. See Figure S9 for numbering scheme.

Bond or angle	Experimental	Calculated
Sb1–Sn1	2.8135(10)	2.868
Sb1–Sn2	2.8804(9)	2.917
Sb1–Sn3	2.8166(10)	2.867
Sb2–Sb3	2.8216(10)	2.845
Sb2–Sn1	2.8402(11)	2.881
Sb2–Sn4	2.8969(11)	2.935
Sb3–Sn3	2.8384(10)	2.881
Sb3–Sn5	2.9058(9)	2.935
Sn1–Sn2	3.2499(10)	3.217
Sn1–Sn4	3.0398(10)	3.029
Sn2–Sn3	3.2720(9)	3.217
Sn2–Sn4	2.9951(9)	3.055
Sn2–Sn5	3.0363(10)	3.055
Sn3–Sn5	3.0063(9)	3.030
Sn4–Sn5	2.9708(10)	3.023
Sb1–Sn1–Sb2	104.43(3)	102.93
Sb1–Sn3–Sb3	103.88(3)	102.95
Sb1–Sn1–Sn4	105.61(3)	108.17
Sb1–Sn3–Sn5	107.09(3)	108.19
Sn1–Sn2–Sn3	88.05(2)	90.94

Table S4. Lowest energy isomers of each structure type of $(Sn_xSb_{8-x})^{2-x}$ (x = 0 – 8). Energies shown are in kJ/mol.

Species	Isomers	
Sb^{2+}	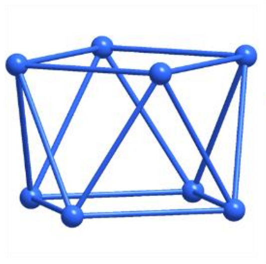	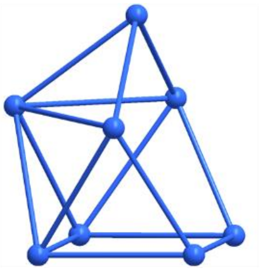
	ST3<br>0	ST1<br>+35.12 kJ/mol

Table S4 (continued).

$(SnSb_7)^{1+}$	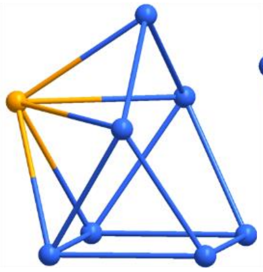	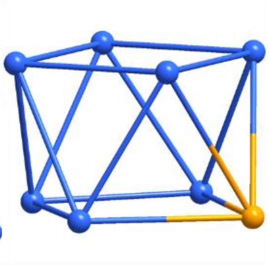		
	ST1<br>0	ST3<br>5.126		
Sn_2Sb_6	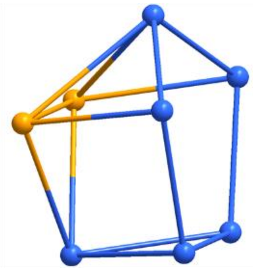	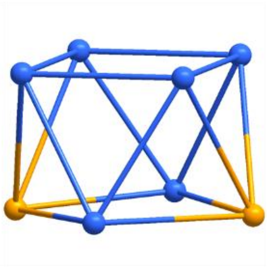	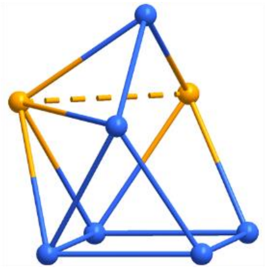	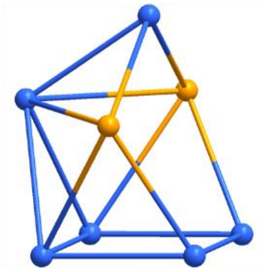
	ST2<br>0	ST3<br>3.821	ST1*<br>8.125	ST1<br>27.55
$(Sn_3Sb_5)^{1-}$	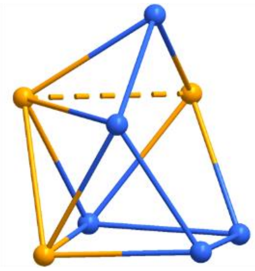	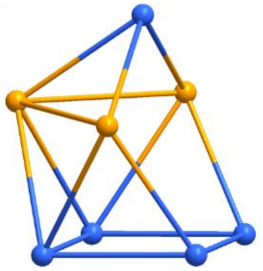	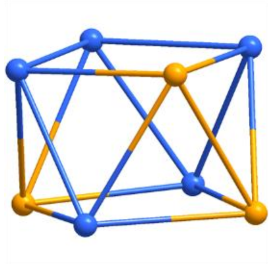	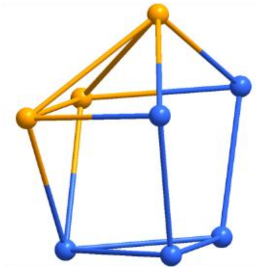
	ST1*<br>0	ST1<br>3.472	ST3<br>16.01	ST2<br>16.94
$(Sn_4Sb_4)^{2-}$	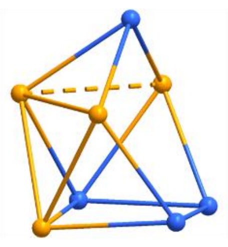	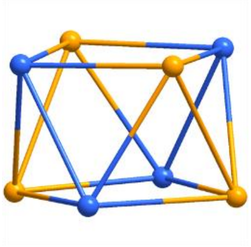	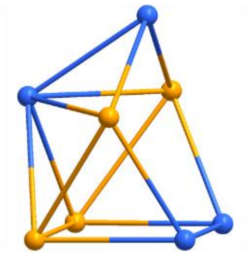	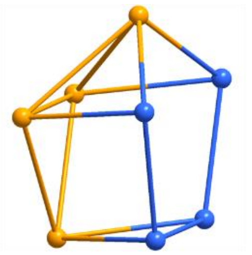
	ST1*<br>0	ST3<br>16.73	ST1<br>23.21	ST2<br>25.06

Table S4 (continued).

$(Sn_5Sb_3)^{3-}$	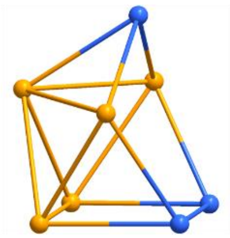	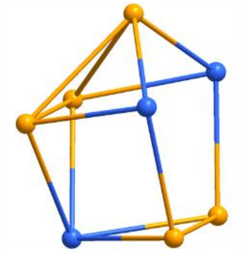	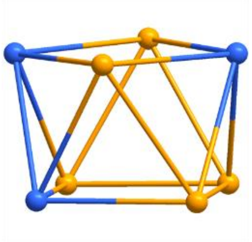	
	ST1	ST2	ST3	
	0	3.251	30.45	
$(Sn_6Sb_2)^{4-}$	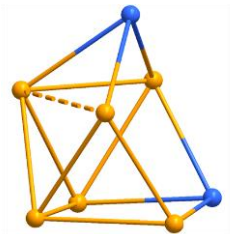	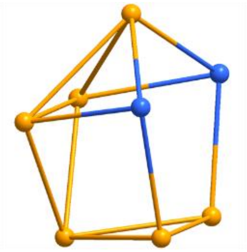	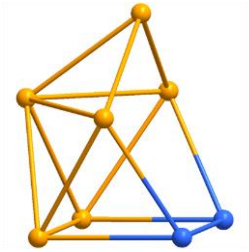	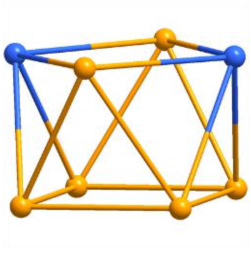
	ST1*	ST2	ST1	ST3
	0	0.7234	5.779	13.28
$(Sn_7Sb_1)^{5-}$	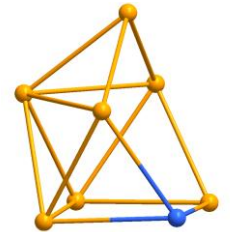	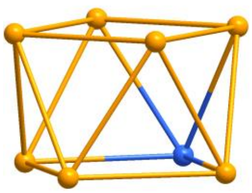	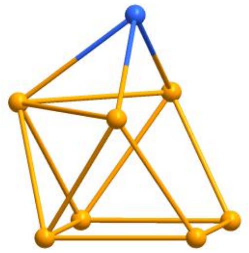	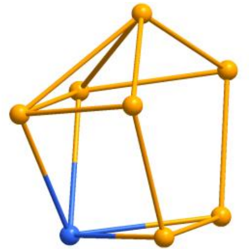
	ST1	ST3	ST1	ST2
	0	7.291	8.811	21.54
Sn_8^{6-}	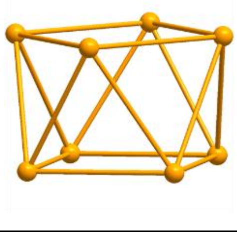	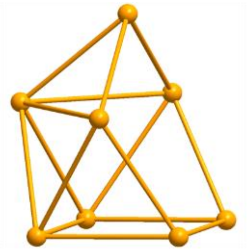		
	ST3	ST1		
	0	9.296		

References

[1] a) G. Sheldrick, *Acta Cryst.* **2008**, *A64*, 112-122; b) G. M. Sheldrick, *Acta Cryst.* **2008**, *A64*, 112-122.

[2] a) O. V. Dolomanov, L. J. Bourhis, R. J. Gildea, J. A. K. Howard, H. Puschmann, *J. Appl. Cryst.* **2009**, *42*, 339-341; b) O. V. Dolomanov, L. J. Bourhis, R. J. Gildea, J. A. K. Howard, H. Puschmann, *J. Appl. Crystallogr.* **2009**, *42*, 339-341.

[3] a) TURBOMOLE, Version 7.4.1. 2019, a development of University of Karlsruhe and Forschungszentrum Karlsruhe GmbH 1989-2007, TURBOMOLE GmbH since 2007, available via https://www.turbomole.org; b) R. Ahlrichs, M. Bär, M. Häser, H. Horn, C. Kölmel, *Chem. Phys. Lett.* **1989**, *162*, 165-169; c) F. Furche, R. Ahlrichs, C. Hättig, W. Klopper, M. Sierka, F. Weigend, *WIREs Comput. Mol. Sci.* **2014**, *4*, 91-100.

[4] B. Metz, H. Stoll, M. Dolg, *J. Chem. Phys.* **2000**, *113*, 2563-2569.

[5] F. Weigend, R. Ahlrichs, *Phys. Chem. Chem. Phys.* **2005**, *7*, 3297-3305.

[6] A. Schäfer, A. Klamt, D. Sattel, J. C. W. Lohrenz, F. Eckert, *Phys. Chem. Chem. Phys.* **2000**, *2*, 2187-2193.

[7] J. Tao, J. P. Perdew, V. N. Staroverov, G. E. Scuseria, *Phys. Rev. Lett.* **2003**, *91*, 146401.

[8] F. Weigend, *Phys. Chem. Chem. Phys.* **2006**, *8*, 1057-1065.
